# Supplementary material for: Heparin-based hydrogel scaffolding alters the transcriptomic profile and increases the chemoresistance of MDA-MB-231 triple-negative breast cancer cells
Source: Biomater Sci. 2020 Feb 13;8(10):2786–96. doi: 10.1039/c9bm01481k (PMC7497406; doi:10.1039/c9bm01481k)
Supplement: Supplementary file 2 [file BM-008-C9BM01481K-s002.zip › Supplementary File 4/HP-B hydrogel + EGF (s)vControl/Pathways/my_analysis.Gsea.1545201170987/HALLMARK_COAGULATION.html]

Details for gene set HALLMARK\_COAGULATION[GSEA]

|  || Dataset | expr.class.cls#HYDEGF\_versus\_CONTROL.class.cls#HYDEGF\_versus\_CONTROL\_repos |
| Phenotype | class.cls#HYDEGF\_versus\_CONTROL\_repos |
| Upregulated in class | CONTROL |
| GeneSet | HALLMARK\_COAGULATION |
| Enrichment Score (ES) | -0.59603935 |
| Normalized Enrichment Score (NES) | -2.5253448 |
| Nominal p-value | 0.0 |
| FDR q-value | 0.0 |
| FWER p-Value | 0.0 |
Table: GSEA Results Summary

  

Fig 1: Enrichment plot: HALLMARK\_COAGULATION      
 Profile of the Running ES Score & Positions of GeneSet Members on the Rank Ordered List

  

| PROBE | DESCRIPTION (from dataset) | GENE SYMBOL | GENE\_TITLE | RANK IN GENE LIST | RANK METRIC SCORE | RUNNING ES | CORE ENRICHMENT || 1 | MST1 | na |  |  | 1218 | 1.564 | -0.0467 | No |
| 2 | CTSK | na |  |  | 1639 | 1.380 | -0.0537 | No |
| 3 | MASP2 | na |  |  | 2367 | 1.160 | -0.0791 | No |
| 4 | F3 | na |  |  | 2957 | 1.016 | -0.0989 | No |
| 5 | SIRT2 | na |  |  | 3643 | 0.877 | -0.1252 | No |
| 6 | MAFF | na |  |  | 4719 | 0.703 | -0.1738 | No |
| 7 | RAPGEF3 | na |  |  | 5160 | 0.628 | -0.1900 | No |
| 8 | TMPRSS6 | na |  |  | 5176 | 0.624 | -0.1840 | No |
| 9 | PLAU | na |  |  | 5696 | 0.547 | -0.2052 | No |
| 10 | MMP15 | na |  |  | 5812 | 0.531 | -0.2055 | No |
| 11 | GP1BA | na |  |  | 6003 | 0.502 | -0.2099 | No |
| 12 | PLG | na |  |  | 6144 | 0.483 | -0.2120 | No |
| 13 | FBN1 | na |  |  | 6535 | 0.433 | -0.2277 | No |
| 14 | CTSH | na |  |  | 6847 | 0.390 | -0.2397 | No |
| 15 | GNB2 | na |  |  | 7318 | 0.327 | -0.2608 | No |
| 16 | FURIN | na |  |  | 7555 | 0.298 | -0.2699 | No |
| 17 | CRIP2 | na |  |  | 7742 | 0.273 | -0.2766 | No |
| 18 | S100A1 | na |  |  | 8257 | 0.215 | -0.3012 | No |
| 19 | HTRA1 | na |  |  | 8915 | 0.133 | -0.3341 | No |
| 20 | BMP1 | na |  |  | 9326 | 0.085 | -0.3547 | No |
| 21 | SERPINB2 | na |  |  | 9903 | 0.024 | -0.3845 | No |
| 22 | WDR1 | na |  |  | 10062 | 0.010 | -0.3927 | No |
| 23 | F8 | na |  |  | 11442 | -0.152 | -0.4632 | No |
| 24 | ANG | na |  |  | 12069 | -0.228 | -0.4935 | No |
| 25 | CFH | na |  |  | 12170 | -0.240 | -0.4961 | No |
| 26 | SH2B2 | na |  |  | 12330 | -0.266 | -0.5015 | No |
| 27 | F2RL2 | na |  |  | 12730 | -0.319 | -0.5189 | No |
| 28 | FYN | na |  |  | 12769 | -0.326 | -0.5174 | No |
| 29 | CTSE | na |  |  | 13010 | -0.343 | -0.5262 | No |
| 30 | ADAM9 | na |  |  | 13107 | -0.354 | -0.5274 | No |
| 31 | CAPN2 | na |  |  | 13169 | -0.364 | -0.5266 | No |
| 32 | LRP1 | na |  |  | 13236 | -0.376 | -0.5260 | No |
| 33 | ISCU | na |  |  | 13541 | -0.419 | -0.5373 | No |
| 34 | LGMN | na |  |  | 13973 | -0.484 | -0.5546 | No |
| 35 | MMP3 | na |  |  | 14191 | -0.504 | -0.5605 | No |
| 36 | F12 | na |  |  | 14317 | -0.523 | -0.5614 | No |
| 37 | PEF1 | na |  |  | 14425 | -0.543 | -0.5610 | No |
| 38 | THBD | na |  |  | 14480 | -0.553 | -0.5579 | No |
| 39 | DUSP14 | na |  |  | 14629 | -0.577 | -0.5593 | No |
| 40 | CAPN5 | na |  |  | 14854 | -0.612 | -0.5644 | No |
| 41 | DPP4 | na |  |  | 15076 | -0.647 | -0.5689 | No |
| 42 | ACOX2 | na |  |  | 15595 | -0.742 | -0.5880 | Yes |
| 43 | A2M | na |  |  | 15638 | -0.749 | -0.5820 | Yes |
| 44 | RABIF | na |  |  | 15681 | -0.758 | -0.5760 | Yes |
| 45 | PDGFB | na |  |  | 15834 | -0.789 | -0.5754 | Yes |
| 46 | MMP11 | na |  |  | 15856 | -0.793 | -0.5678 | Yes |
| 47 | CFD | na |  |  | 15870 | -0.795 | -0.5599 | Yes |
| 48 | ANXA1 | na |  |  | 15923 | -0.806 | -0.5538 | Yes |
| 49 | RGN | na |  |  | 16272 | -0.891 | -0.5623 | Yes |
| 50 | CASP9 | na |  |  | 16279 | -0.893 | -0.5530 | Yes |
| 51 | GSN | na |  |  | 16299 | -0.897 | -0.5442 | Yes |
| 52 | CSRP1 | na |  |  | 16302 | -0.898 | -0.5345 | Yes |
| 53 | SERPINA1 | na |  |  | 16347 | -0.909 | -0.5270 | Yes |
| 54 | PRSS23 | na |  |  | 16650 | -0.985 | -0.5320 | Yes |
| 55 | TIMP1 | na |  |  | 16662 | -0.990 | -0.5219 | Yes |
| 56 | USP11 | na |  |  | 16668 | -0.992 | -0.5113 | Yes |
| 57 | PREP | na |  |  | 16698 | -0.999 | -0.5020 | Yes |
| 58 | CFB | na |  |  | 16882 | -1.049 | -0.5002 | Yes |
| 59 | PLAT | na |  |  | 16888 | -1.050 | -0.4890 | Yes |
| 60 | ARF4 | na |  |  | 17138 | -1.125 | -0.4898 | Yes |
| 61 | C3 | na |  |  | 17200 | -1.144 | -0.4806 | Yes |
| 62 | C2 | na |  |  | 17358 | -1.202 | -0.4757 | Yes |
| 63 | C1R | na |  |  | 17523 | -1.268 | -0.4705 | Yes |
| 64 | KLF7 | na |  |  | 17603 | -1.294 | -0.4606 | Yes |
| 65 | GNG12 | na |  |  | 17703 | -1.333 | -0.4513 | Yes |
| 66 | RAC1 | na |  |  | 17921 | -1.452 | -0.4468 | Yes |
| 67 | MMP1 | na |  |  | 17947 | -1.463 | -0.4322 | Yes |
| 68 | CFI | na |  |  | 18016 | -1.493 | -0.4196 | Yes |
| 69 | APOC1 | na |  |  | 18177 | -1.597 | -0.4106 | Yes |
| 70 | S100A13 | na |  |  | 18364 | -1.725 | -0.4015 | Yes |
| 71 | LTA4H | na |  |  | 18508 | -1.843 | -0.3890 | Yes |
| 72 | CTSB | na |  |  | 18524 | -1.856 | -0.3696 | Yes |
| 73 | APOA1 | na |  |  | 18534 | -1.861 | -0.3498 | Yes |
| 74 | SPARC | na |  |  | 18543 | -1.861 | -0.3300 | Yes |
| 75 | CLU | na |  |  | 18586 | -1.909 | -0.3115 | Yes |
| 76 | TIMP3 | na |  |  | 18706 | -2.053 | -0.2954 | Yes |
| 77 | MSRB2 | na |  |  | 18757 | -2.122 | -0.2749 | Yes |
| 78 | LAMP2 | na |  |  | 18797 | -2.190 | -0.2531 | Yes |
| 79 | CTSO | na |  |  | 18944 | -2.443 | -0.2342 | Yes |
| 80 | ITGA2 | na |  |  | 18964 | -2.492 | -0.2081 | Yes |
| 81 | DUSP6 | na |  |  | 18966 | -2.496 | -0.1810 | Yes |
| 82 | ITGB3 | na |  |  | 18981 | -2.535 | -0.1542 | Yes |
| 83 | TFPI2 | na |  |  | 18994 | -2.568 | -0.1269 | Yes |
| 84 | CD9 | na |  |  | 19017 | -2.631 | -0.0995 | Yes |
| 85 | PECAM1 | na |  |  | 19026 | -2.661 | -0.0710 | Yes |
| 86 | C1S | na |  |  | 19156 | -3.351 | -0.0413 | Yes |
| 87 | PROS1 | na |  |  | 19188 | -3.994 | 0.0005 | Yes |
Table: GSEA details [plain text format]

  

Fig 2: HALLMARK\_COAGULATION      
 Blue-Pink O' Gram in the Space of the Analyzed GeneSet

  

Fig 3: HALLMARK\_COAGULATION: Random ES distribution      
 Gene set null distribution of ES for **HALLMARK\_COAGULATION**
